# Association of Spinal Alignment and Abdominal Circumference with Sarcopenia Status and Fall Risk in Patients with Osteoporosis: A Retrospective Study

**DOI:** 10.3390/nu15112571

**Published:** 2023-05-31

**Authors:** Takashi Nagai, Makoto Miyagami, Ichiro Okano, Shota Nakamura, Yuichiro Okazaki, Keizo Sakamoto, Fumihito Kasai, Yoshifumi Kudo, Nobuyuki Kawate

**Affiliations:** 1 Department of Rehabilitation Medicine, Showa University School of Medicine, Tokyo 142-866, Japan; fumihito@med.showa-u.ac.jp (F.K.); kawate@med.showa-u.ac.jp (N.K.); 2 Department of Orthopedic Surgery, Showa University School of Medicine, Tokyo 142-866, Japan; miyagamism@gmail.com (M.M.); ichiro.okano.e31@gmail.com (I.O.); s_n_s7pc@yahoo.co.jp (S.N.); s14-018oy@nms.ac.jp (Y.O.); kz-saka@mug.biglobe.ne.jp (K.S.); kudo_4423@med.showa-u.ac.jp (Y.K.)

**Keywords:** osteoporosis, sarcopenia, obesity, abdominal circumference, fall risk

## Abstract

Since vertebral kyphosis and abdominal circumference are thought to influence sarcopenia and fall risk in osteoporosis, we evaluated sarcopenia and fall risk in patients with different measurements of abdominal circumference and sagittal longitudinal axis (SVA). In this post hoc study, 227 patients aged 65 years or more who visited an outpatient osteoporosis clinic were included in the analysis. Sarcopenia was determined from lean body mass, grip strength, and walking speed by dual energy X-ray absorptiometry; SVA (median 40 mm) and abdominal circumference (median 80 cm) were compared between the four groups, each divided into two groups. Nutritional management, falls, and fall anxiety scores were also examined. The incidence of sarcopenia was significantly increased in those with abdominal circumference < 80 cm in both the SVA < 40 mm and SVA ≥ 40 mm groups (*p* < 0.05). Nonetheless, the fall scores of those with SVA < 40 mm were lower than those of individuals with SVA ≥ 40 mm (*p* < 0.01). Based on the results of this study, SVA and abdominal circumference values may predict the risk of sarcopenia and falls. More research is needed before our results can be translated into clinical practice.

## 1. Introduction

The prevalence of osteoporosis worldwide is 18.3% (95% confidence interval 16.2–20.7), and 23.1% of women (95% confidence interval 19.8–26.9) [1]. The number of older people in Japan and the incidence of osteoporosis are increasing [2,3]. In this context, it is necessary to prevent falls because resulting femoral neck and vertebral body compression fractures can lead to bed confinement, bedridden pressure ulcers, and the reduction in an individual’s capacity to perform activities of daily living (ADL) [4,5]. Therefore, to prevent fractures, it is important to increase bone density and prevent falls. Muscle mass decreases with age [6], which also contributes to the risk of falls [7]. The decrease in skeletal muscle mass and muscle strength with aging is called sarcopenia, which can be determined by the muscle mass of the limbs, walking speed, and grip strength [8,9]. Moreover, decreased skeletal muscle mass also affects spinal alignment [10]. Some individuals may not be aware that they are at a high risk of falling. Thus, it is important to identify and alert individuals who are likely to fall at an early stage.

Aging causes progressive retroversion of the pelvis [11]. Weight gain causes kyphotic deformity of the spine [12]. Owing to either deformity, hip and knee joints compensate for each other, resulting in flexion contractures of the hip and knee [13]. A posterior pelvic tilt increases the risk of falls [10]. Thus, measuring the global alignment of the spine allows physicians to identify individuals at a high risk of falling [14].

Obesity is also associated with falls and is considered a risk factor for fractures and other injuries [15]. Visceral fat volume can be estimated via abdominal circumference measurement and is thought to be associated with metabolic syndrome [16]. In addition, an increased abdominal circumference may lead to kyphosis of the lumbar spine. The thoracic spine maintains anterior–posterior balance in those with a kyphosis deformity [17]. Therefore, a greater abdominal circumference increases the risk of falling [18].

In osteoporosis, we assumed that kyphosis may be associated with a short stature, making the patient less likely to experience sarcopenia but more likely to fall because of poor balance. Therefore, a large abdominal circumference, resulting from overnutrition, may make the patient susceptible to falls. Thus, this study aimed to evaluate the risk of sarcopenia and falls in patients with osteoporosis with differing abdominal circumference and sagittal vertical axis (SVA) values.

## 2. Materials and Methods

This retrospective study complied with the Declaration of Helsinki guidelines for research on human subjects. The study was approved by the Showa University’s ethics committee on research involving human subjects (Approval No. 21-194-A). In addition, informed consent in this study was secured by adopting an opt-out consent method at the ethics committee mentioned earlier. Patient anonymity was preserved.

### 2.1. Patients

**Figure 1:**
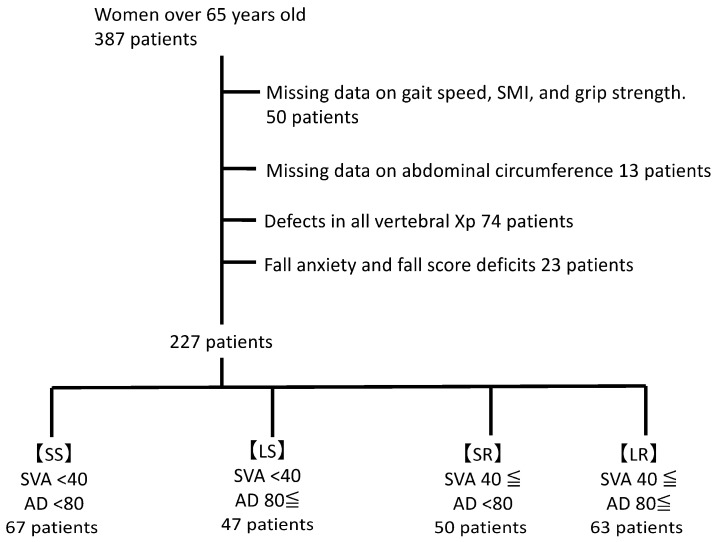
Patient selection and group allocation. Of the 387 women aged 65 years or more were considered, 227 were included in the analysis after excluding 160 women with missing data. Patients were divided into four groups according to median abdominal circumference and SVA measurements.

This study considered data from patients aged 65 years or more who were admitted to the Higashi Hospital, Showa University Hospital’s (Tokyo, Japan) outpatient osteoporosis clinic from January 2017 to December 2022. Patients with insufficient available data were excluded from the analysis. Patient selection and group allocation strategies are shown in Figure 1.

### 2.2. Study Design

Bone density was measured using dual-energy X-ray absorptiometry (DXA; Discovery DXA System, Hologic, Inc., Marlborough, MA, USA), and skeletal muscle mass was assessed using the SMI. In addition, lean body mass and adiposity of the trunk were measured based on DXA results.

### 2.3. Evaluation of Hand Grip Strength

Grip strength was measured using a Smedley hand dynamometer (MY-2080; Matsumiya Medical Seiki Corporation, Tokyo, Japan). Grip strength was measured three times in the sitting position, following previous studies [10,19,20]. The median value of each side represented each patient’s grip strength. The largest median value reported for the left or right side was considered an individual’s overall grip strength.

### 2.4. Assessment of Fall Risk

The following five data items were used to assess fall risk: 1. whether the patient had fallen in the past year; 2. whether the patient thought their walking speed had recently decreased; 3. whether the patient used a cane; 4. whether the patient thought their back had recently rounded; and 5. whether the patient took more than five medications. These data were collected through interview interviews during the consultation [21]. Item 1 scored five points, and items 2–5 scored two points each for a total score of 13 points. In this falls score, five of the factors associated with falls that were significantly different by logistic regression analysis were selected, and the score was calculated by integerizing the fall risk for each. Using a score of 6 as the cutoff, the sensitivity and specificity were 0.68 and 0.70, respectively, with 28% falling within 6 months for a score of 6 or higher and 7% falling within 6 months for a score of less than 6. Therefore, a fall score of 6 or higher was considered high risk for falls [20,21,22]. Fall anxiety was assessed via interview assessment, using a 2-point scale with “always anxious about falling” or “sometimes anxious about falling” considered indicative of having fall anxiety, and “not anxious about falling”, indicating no fall anxiety.

### 2.5. Sarcopenia

Sarcopenia is diagnosed based on the SMI, which is calculated by dividing the muscle mass (kg) of the extremities by the square of the stature of the patient (m) [8]. We used the 2019 Asian Sarcopenia Working Group criteria for the enrolled participants with sarcopenia [23]. Based on DXA data, the criteria classified individuals by height-adjusted muscle mass < 5.4 kg/m^2^, walking speed < 1.0 m/s, and grip strength < 18 kg.

The Charlson comorbidity index (Controlling Nutritional Status (CONUT)) indicates nutritional status. The CONUT is scored according to the total cholesterol level, lymphocyte count, and serum albumin level [24]. To determine the level of nutrition, 0–1 point was considered normal; 2–4, mild; 5–8, moderate; and 9–12, severe. The CONUT correlates with the results of the subjective global assessment and the full nutritional assessment [24,25].

Furthermore, abdominal circumference and vertebral kyphosis were assessed. The shortest distance between the vertical line passing through the center of the seventh cervical vertebral body and the upper margin of the posterior wall of the first sacral vertebra (i.e., SVA) was measured using lateral spine radiographs (Medical Systems USA, Inc., version 4.1.50107, New York, NY, USA). Radiographs were routinely obtained at the outpatient clinic in the standing position [14]. Moreover, for abdominal muscle deflection, a vertical line was drawn through the apex of the abdominal muscles and the upper edge of the S1 posterior wall using lateral spine radiographs. The shortest distance between these two lines, the sacral–abdominal wall distance (SAD), was measured. If the 1st sacral vertebra was difficult to determine in the transitional vertebrae, the 25th vertebra from the first cervical vertebra was used as the first sacral vertebra. For simple lateral radiographs of the spine, the patient stood in a natural standing position with hands touching the shoulders from the front so that the spine did not overlap with the upper extremities. The patient put on an examination gown provided by the hospital prior to the examination. Consequently, the abdominal circumference was measured after expiration, with the patient standing in a natural position with both legs together at the level of the umbilicus to avoid abdominal compression [26]. One measurement was taken.

### 2.6. Patient Groups

Patients were grouped according to the SVA and abdominal circumference values. First, the patients were classified into two groups based on the median SVA (40 mm). Next, they were further classified into four groups according to the median SVA and median abdominal circumference (80 cm) values as follows: short-straight back (SS), SVA < 40 mm and abdominal circumference < 80 cm; long-straight back (LS), SVA < 40 mm and abdominal circumference ≥ 80 cm), short-round back (SR), SVA ≥ 40 mm and abdominal circumference < 80 cm; and long-round back (LR), SVA ≥ 40 mm and abdominal circumference ≥ 80 cm).

### 2.7. Data Analysis and Statistical Methods

The Student’s *t*-test was used to compare continuous variables with normal distributions, and the Mann–Whitney *U* test was used to compare continuous variables with non-normal distributions. The Kruskal–Wallis test was used to assess the four groups. In addition, tests of the percentage of sarcopenia, fall score > 6, fall anxiety, and vertebral fracture history between the two and four groups were performed using the χ^2^ test. Multiple logistic regression analysis was performed for high fall risk (fall score > 6 or 6 points) as the objective variable and sarcopenia, SVA, and abdominal circumference as explanatory variables. Two-sided *p* < 0.05 was considered to indicate statistical significance. Analyses were performed using StatFlex (ver. 7.0.8; Igaku Tokei Kenkyujo. Inc., Ube, Japan) software. The sample size test was performed using G*Power (ver. 3.1.9.6; Heinrich Heine University, Düsseldorf, Germany).

### 2.8. Number of Samples Required

Setting alpha error as 0.05, power (1-beta error) as 0.95, and effect size as 0.5, the required number of samples was 210.

## 3. Results

### 3.1. Baseline Patient Characteristics and Group Allocation

Baseline characteristics of patients were extracted from existing data. In total, 387 patients aged 65 years or older who visited the department’s osteoporosis outpatient clinic were included in the study. Among them, the following were excluded: 50 patients with missing data on gait speed, grip strength, and muscle mass of the extremities; 13 with missing data on the abdominal circumference; 74 without radiographic images of the entire spine; and 23 with missing fall-related data. Therefore, data from 227 patients was included in the final analysis. The mean age of the 227 included patients was 77.5 ± 7.2 (mean ± standard deviation) years. Patient characteristics are summarized in Table 1.

Characteristics of patients with SVA values greater and lesser than 40 mm and abdominal circumference values greater and lesser than 80 cm are summarized in Table 2. SVA values were <40 mm in 114 patients and ≥40 mm in 113 patients. Furthermore, 117 patients had an abdominal circumference value < 80 cm, while the abdominal circumference was ≥80 cm in 110 patients. There were 67, 47, 50, and 63 patients in the SS, LS, SR, and LR groups, respectively.

### 3.2. Between-Group Comparisons

#### 3.2.1. Participant Numbers

The SVA was <40 mm in 114 patients and ≥40 mm in 113 patients. In total, 117 patients had an abdominal circumference < 80 cm, and 110 patients had an abdominal circumference > 80 cm. A total of 67, 47, 50, and 63 patients were classified into the SS, LS, SR, and LR groups, respectively (Table 2).

#### 3.2.2. Body Composition

##### SMI

The SMI values of the SS group significantly differed from those of the LS and LR groups (*p* < 0.001). Furthermore, the SMI values of the SR group significantly differed from those of the LS and LR groups (*p* < 0.001) (Table 2).

##### Trunk Fat

Trunk fat percentage did not differ between the SVA ≥ 40 mm and SVA < 40 mm groups (*p* = 0.97). However, within each of the SVA ≥ 40 mm and SVA < 40 mm groups, it was significantly higher in patients with abdominal circumference ≥ 80 cm (*p* < 0.001) (Table 2).

#### 3.2.3. HbA1c

HbA1c was significantly higher in the SVA ≥ 40 mm group than in the SVA < 40 mm group (*p* < 0.01), and abdominal circumference ≥ 80 cm was significantly higher than 80 cm in the SVA ≥ 40 mm group (*p* < 0.01) (Table 2).

#### 3.2.4. Sarcopenia

**Figure 2:**
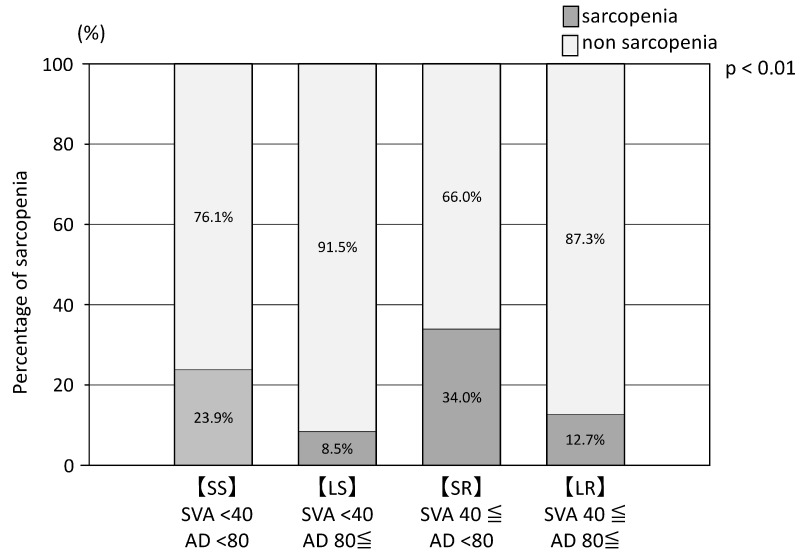
Proportion of sarcopenia in SS (SVA < 40 and AD < 80), LS (SVA < 40 and AD80≤), SR (SVA 40≤ and AD < 80), and LR (SVA 40≤ and AD 80≤) groups. χ-square test showed that in AD < 80 the proportion of sarcopenia was higher in AD < 80 (*p* < 0.01).

There was no group difference in the incidence of sarcopenia when patients with SVA values ≥ 40 mm and <40 mm were compared. However, the incidence of sarcopenia was significantly elevated among those with abdominal circumference values < 80 cm, a finding that was independent of SVA (Figure 2, Table 2).

#### 3.2.5. Bone Mineral Density

Bone mineral density values of the L2–4 lumbar vertebrae were greatest among those with an SVA value ≥ 40 mm and an abdominal circumference ≥ 80 cm. By contrast, bone mineral density was lowest among those with an SVA value < 40 mm and an abdominal circumference < 80 cm. The bone density of the femoral neck of patients of all four groups did not differ significantly (Table 2).

#### 3.2.6. CONUT

For the SVA < 40 mm group, the CONUT scores of individuals with an abdominal circumference <80 cm was higher than those of individuals with an abdominal circumference ≥ 80 cm. Among those with SVA values ≥ 40 mm, no CONUT score differences were observed based on abdominal circumference values ≥ or <80 cm (Table 2).

#### 3.2.7. Fall Score and Fall Anxiety

**Figure 3:**
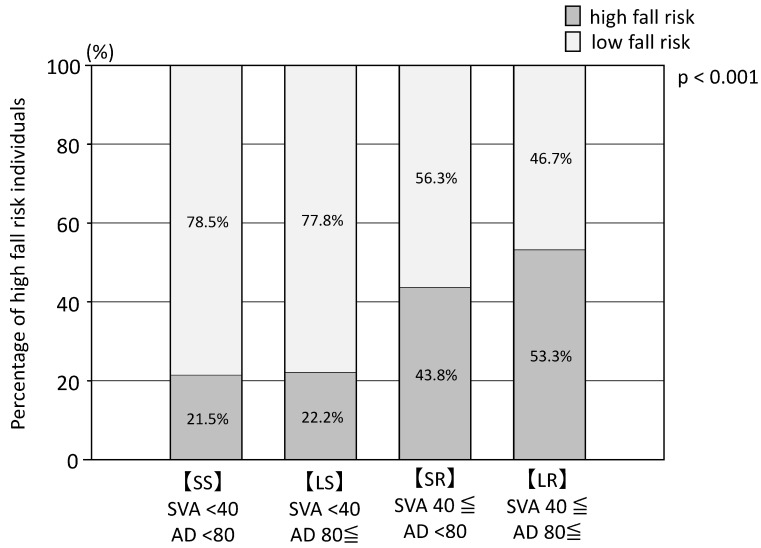
Proportion of fall risk (fall score ≥ 6 points) in SS (SVA < 40 and AD < 80), LS (SVA < 40 and AD 80≤), SR (SVA 40≤ and AD < 80), and LR (SVA 40≤ and AD 80≤) groups. The χ-square test showed that the rate of fall risk was higher for SVA40≤ (*p* < 0.001).

The SS and LS groups had lower fall scores than the LR and SR groups. The participants with fall scores ≥ 6 were 22% (14/65) for the SS, 44% (21/48) for the SR, 22% (10/45) for the LS, and 53% (32/60) for the LR group. Falls scores were significantly higher for participants with SVA values > 40 mm than for those with SVA values < 40 mm (Figure 3). Fall anxiety was reported in 29 of 65 (45%), 26 of 46 (57%), 9 of 35 (20.5%), and 39 of 62 (63%) patients of the SS, SR, LS, and LR groups, respectively (Table 2).

### 3.3. Fall Risk Factors

Multiple logistic regression analysis was performed for a fall score of 6 or higher as the objective variable and age-adjusted sarcopenia, SVA, and abdominal circumference as dependent variables. We found that sarcopenia (*p* < 0.01), SVA (*p* < 0.001), and abdominal circumference (*p* < 0.05) were independent factors (Table 3).

## 4. Discussion

This study investigated the presence of sarcopenia using global spinal alignment and abdominal measurements. We predicted that the kyphosis deformity of the spine would reduce abdominal and back muscle mass [27], making the patient more prone to sarcopenia. However, even without a kyphosis deformity, a small abdominal circumference was found to predispose to sarcopenia. In Japan, because of the mild prevalence and degree of obesity, an abdominal circumference of 90 cm, which approximates the visceral fat area, indicates the risk of fat-related diseases and is the standard for obesity [28]. In this study, the patients were classified by 80 cm abdominal circumference using median values, and thus, they were not classified by obesity. Notably, increased abdominal circumference is also associated with decreased cardiorespiratory function [29] and decreased grip strength and walking speed [30]. Moura et al. studied 10-year mortality in women aged 60 years and older, classified by 88 cm and 77.8 cm abdominal circumferences, and observed that both reported higher cardiovascular mortality [31]. Even when classified by the median, we thought that they could observe differences between abdominal circumferences greater than 80 cm and less than 80 cm. Although back extensor strength correlates with spinal alignment, it has been reported that limb skeletal muscle mass, used in diagnosing sarcopenia, does not correlate with spinal alignment [32]. By contrast, the skeletal muscle mass of the trunk is negatively correlated with SVA [27], and strong back pain is associated with greater sarcopenia and SVA [33,34]. In this study, spinal alignment and abdominal circumference were incorporated for comparison, and the characteristics of each group were identified.

In this study, the age of the patients in the LR group was the highest, followed by those in the SR, LS, and SS groups, respectively. In addition, the proportion of vertebral fractures increased with age, suggesting an increase in SVA. Furthermore, no difference in height was observed between the SS and LS groups or the SR and LR groups; however, significant differences were observed in the percentage of SMI and sarcopenia between the SS and LS groups and between the SR and LR groups. When the patients were classified into those with an abdominal circumference of 80 cm or more and those with an abdominal circumference of less than 80 cm, the percentage of sarcopenia was higher in the SS and SR groups with an abdominal circumference of less than 80 cm. The diagnostic criteria for sarcopenia classify the lean mass of the extremities as muscle mass, and lighter body weight tends to indicate decreased muscle mass, thus increasing the rate of sarcopenia. Yoo et al. found that in elderly Koreans, the greater the body fat percentage and abdominal circumference, the lesser the sarcopenia severity [35]. Interestingly, our results were similar. Furthermore, CONUT scores, a measure of nutrition, were significantly higher in patients with SVA > 40 mm than in those with SVA < 40 mm (*p* < 0.001). CONUT is a method of assessing nutritional status based on serum albumin, total cholesterol, and lymphocyte counts and is a potential predictive marker of postoperative complications in patients with hip fractures [36]. In our results, lighter weight was associated with a higher CONUT score. The lack of difference in CCI suggests that the presence or absence of pre-existing conditions did not have much effect on the CONUT score. In addition, CONUT includes dietary intake and calories consumed [37]; therefore, moderate dietary intake is necessary to prevent sarcopenia.

In this study, HbA1c levels were significantly higher in those with SVA ≥ 40 mm than in those with SVA < 40 mm. Furthermore, in the group with SVA ≥ 40 mm, HbA1c was significantly higher in those with abdominal circumference ≥ 80 cm than those with abdominal circumference < 80 cm. However, there was no difference in the HbA1c levels according to abdominal circumference for those with an SVA < 40 mm. Differences in fall anxiety among the patients may have led to differences in the amount of exercise performed because of the limited amount of usual activity or hesitation in going out. More than 40% of the patients in the SS group and more than half in the SR and LR groups experienced fall anxiety. By contrast, more than 40% of the patients in the SR and LR groups had fall risk scores of ≥6, while 21.5% and 22.2% of the patients in the SS and LS groups, respectively, had scores ≥ 6. The SS group had greater fall anxiety relative to fall risk, for which low SMI and decreased muscle mass may have been contributing factors. Moreover, other factors, such as the center-of-gravity and balance, may be involved; however, these were not examined in this study. The fall risk score used in this study was based on five items, one of which was a question about spinal kyphosis, which was subjective to the patient. The SVA measured in this study was an objective value, and its division by median value allowed for a relative judgment. Nevertheless, we cannot deny the possibility that both influenced the results. In the future, we plan to clarify whether subjective judgments and objective SVA influenced each other by examining the detailed item by-item fall scores.

In addition, a larger SVA was correlated with a higher percentage of vertebral fractures in this study. Moreover, crushing the vertebral body may have increased kyphotic deformity and SVA. For instance, for patients with SVA < 40 mm, a greater proportion of vertebral fractures occurred with greater abdominal circumference. Thus, as body weight increases, bone fragility increases owing to the replacement of osteoblasts by adipocytes in the bone marrow and the increased inflammation present in obese individuals [38]. Furthermore, Kim et al. reported that a higher body fat percentage and larger waist circumference resulted in more vertebral fractures [39]. In this study, there was also a significant difference in trunk adiposity between the SS and LS groups, which may have resulted in the difference in vertebral fracture incidence.

Moreover, we expected that a strong kyphotic spine deformity would result in a short stature, making the patient less likely to develop sarcopenia but more likely to have poor balance and falls. In addition, we expected that a large abdominal circumference would result in overfeeding and increase the incidence of falls. The multiple logistic regression analysis showed that overall, greater abdominal circumference was a risk factor for falls. Nevertheless, a larger abdominal circumference, even with a rounded back, was associated with less sarcopenia, and a larger abdominal circumference but smaller SVA was associated with a lower risk of falls.

The limitation of this study was that only patients attending an urban hospital were included, which may not represent populations from all regions. In addition, the study included patients with osteoporosis, which may have originally attracted cases who were aware of fractures and falls. Furthermore, it was a retrospective study and includes cases already being treated for osteoporosis. The nature of our study precludes any consideration of causality. Therefore, the possibility that osteoporosis medications may have influenced the data cannot be ruled out. Finally, the abdominal circumference was measured at the time of the examination, and values may differ with a full stomach, fasting, or constipation.

Furthermore, we did not investigate the decrease in abdominal muscle mass or atrophy of the abdominal muscles in this study. We believe that a decrease in abdominal muscle mass and power is related to abdominal circumference, spinal alignment, and sarcopenia, which we are currently investigating.

## 5. Conclusions

We examined sarcopenia status in osteoporotic women aged ≥65 years with differing median SVA and abdominal circumference values. For both SVA < 40 mm and SVA > 40 mm groups, sarcopenia incidence increased among those with abdominal circumference values < 80 cm. The kyphosis deformity of the spine reduces abdominal and back muscle mass and may predispose to sarcopenia. However, even without kyphosis, a small abdominal circumference was found to be associated with sarcopenia. More research is needed before our results can be translated into clinical practice.

## Figures and Tables

**Table 1:** Patient characteristics.

Age (years), Median [IQR]	78.0 [72.0–82.0]
BMI (kg/m^2^), mean (SD)	21.5 (3.6)
<18.5, *n* (%)	46 (20.3)
18.5–25, *n* (%)	148 (65.2)
25–30, *n* (%)	28 (12.3)
>30, *n* (%)	5 (2.2)
Height (cm), median [IQR]	151.1 [146.2–154.6]
Weight (kg), median [IQR]	48.7 [42.0–53.7]
Anti-osteoporotic drugs	
Any, *n* (%)	214 (94)
SERM, *n* (%)	54 (23.8)
Bisphosphonate, *n* (%)	94 (41.4)
Denosumab, *n* (%)	43 (18.9)
Teriparatide, *n* (%)	7 (3.1)
CCI (points), median [IQR]	0.0 [0.0–2.0]
SMI (kg/m^2^)	5.8 (0.7)
Albumin (g/dL), median [IQR]	4.1 [4.0–4.3]
Serum calcium level (mg/dL), median [IQR]	9.4 [9.2–9.7]
Serum creatinine level (mg/dL), median [IQR]	0.68 [0.59–0.77]
eGFR, mean (SD)	63.9 (16.1)
T-col (mg/dL), mean (SD)	215.7 (30.4)
HDL-C (mg/dL), mean (SD)	69.9 (16.5)
LDL-C (mg/dL), mean (SD)	119.4 (27.0)
PTH-intact (pg/mL), median [IQR]	30.0 [24.0–39.8]
HbA1c (%), median [IQR]	5.8 [5.5–6.0]
25(OH)D (ng/mL), mean (SD)	20.6 (7.7)
Bone mineral density (spine; g/cm^2^), median [IQR]	0.789 [0.689–0.884]
Bone mineral density (hip neck; g/cm^2^), mean (SD)	0.528 (0.091)
Bone mineral density (hip total; g/cm^2^), mean (SD)	0.641 (0.098)
Bone mineral density (hip trochanter; g/cm^2^), mean (SD)	0.506 (0.082)
PT (°), median [IQR]	19.0 [13.0–28.0]
SVA (mm), median [IQR]	39.7 [17.0–78.8]
Trunk fat (%), mean (SD)	29.8 (8.2)
Hand grip (kg), mean (SD)	18.7 (4.7)
Abdominal circumference (cm), mean (SD)	79.9 (10.3)
SAD (mm), median [IQR]	158.1 [144.0–178.8]
CONUT score (points), median [IQR]	1.0 [0.0–1.0]
Frailty (points), median [IQR]	1.0 [0.0–2.0]

Equivariance is expressed as mean (standard deviation), and a lack of equivariance is expressed as median (interquartile ranges). SD: standard deviation, n: number, IQR: interquartile range, BMI: body mass index, CCI: Charlson Comorbidity index, SMI: skeletal muscle mass index, eGFR: estimated glomerular filtration rate, T-col: total lipoprotein cholesterol, LDL-col: low-density lipoprotein cholesterol, HDL-col: high-density lipoprotein cholesterol, PTH: parathyroid hormone, HbA1c: hemoglobin A1c, 25(OH)D: 25-hydroxyvitamin D, PT: pelvic tilt, SVA: sagittal vertical axis, SAD: sacral vertebra–abdominal wall distance, CONUT: Controlling Nutritional Status.

**Table 2:** Characteristics of patients with SVA values greater and lesser than 40 mm and abdominal circumference greater and lesser than 80 cm.

	SVA < 40			SVA ≥ 40				
	Abdominal Circumference <80 cm	Abdominal Circumference ≥80 cm	*p*-Value	Abdominal Circumference <80 cm	Abdominal Circumference ≥80 cm	*p*-Value	Comparison among All Four Groups	Comparison of SVA < 40 mm and SVA ≥ 40 mm Groups
	[SS]	[LS]		[SR]	[LR]			
Age (years), mean (SD)	73.7 (5.9)	75.7 (6.7)	0.12	79.6 (6.6)	81.1 (7.1)	0.14	*p* < 0.001	*p* < 0.001
BMI (kg/m^2^), mean (SD)	19.5 (2.5)	23.5 (2.6)	<0.001	19.2 (2.6)	24.0 (3.4)	<0.001	*p* < 0.001	*p* = 0.22
Height (cm), mean (SD)	152.0 (5.6)	151.7 (5.8)	0.71	149.7 (5.6)	148.5 (6.6)	0.45	*p* < 0.05	*p* < 0.01
Weight (kg), mean (SD)	45.0 (6.0)	54.1 (7.3)	<0.001	43.1 (5.8)	53.0 (8.3)	<0.001	*p* < 0.001	*p* = 0.63
SMI (kg/m^2^), mean (SD)	5.5 (0.6)	6.1 (0.7)	<0.001	5.6 (0.6)	6.1 (0.7)	<0.001	*p* < 0.001	*p* = 0.09
Percentage of sarcopenia, *n* (%)	16 (23.9)	4 (8.5)	0.03	17 (34%)	8 (12.7%)	<0.01	*p* < 0.01	*p* = 0.39
CCI (points), mean (SD)	0.8 (1.2)	0.7 (1.4)	0.27	1.0 (1.4)	1.1(1.5)	0.68	0.38	0.11
Bone mineral density, spine (g/cm^2^), mean (SD)	0.735 (0.143)	0.803 (0.165)	0.02	0.808 (0.159)	0.856 (0.182)	0.28	*p* < 0.001	*p* < 0.001
Bone mineral density, hip neck (g/cm^2^), mean (SD)	0.520 (0.077)	0.542 (0.115)	0.24	0.516 (0.082)	0.535 (0.089)	0.24	*p* = 0.41	*p* = 0.82
HbA1c (%), mean (SD)	5.8 (0.6)	5.8 (0.5)	0.74	5.9 (0.7)	6.3 (1.2)	<0.001	*p* < 0.001	*p* < 0.01
CONUT score (points), mean (SD)	0.7 (0.9)	0.4 (0.6)	<0.05	1.2 (1.1)	1.0 (1.0)	0.49	*p* < 0.01	*p* < 0.001
CONUT 0–1 (point), *n* (%)	56 (84)	44 (94)		31 (62)	41 (65)			
CONUT 2–4 (points), *n* (%)	11(16)	3 (6)		18 (36)	20 (32)			
CONUT 5–8 (points), *n* (%)	0(0)	0 (0)		0 (0)	0 (0)			
CONUT unclear, *n* (%)	0(0)	0 (0)		1 (2)	2 (3)			
Fall down score, mean (SD)	3.1 (3.1)	3.3 (3.4)	0.77	5.1 (3.4)	5.8 (3.5)	0.26	*p* < 0.001	*p* < 0.001
Fall scores > 6, *n* (%)	14 (21.5)	10 (22.2)	0.93	21 (43.8)	32 (53.3)	0.32	*p* < 0.001	*p* < 0.001
Eyes open, one leg standing time (second), mean (SD)	13.0 (4.0)	11.4 (5.0)	0.05	10.5 (5.4)	6.5 (5.7)	<0.001	*p* < 0.001	*p* < 0.001
Grip power, mean (SD)	20.1 (4.1)	19.7 (4.8)	0.88	17.6 (5.0)	17.5 (4.4)	0.83	*p* < 0.01	*p* < 0.001
Sense of insecurity about falling, *n* (%)	29 (44.6)	9 (20.5)	<0.01	26 (56.5)	39 (62.9)	0.50	*p* < 0.001	*p* < 0.001
Vertebral fracture history, *n* (%)	23 (34.3)	27 (57.4)	0.01	37 (74.0)	40 (63.5)	0.23	*p* < 0.001	*p* < 0.001
PT, mean (SD)	16.3 (10.2)	16.9 (8.0)	0.41	22.5 (11.8)	28.2 (10.5)	0.01	*p* < 0.001	*p* < 0.001
Trunk fat (%), mean (SD)	26.6 (6.7)	34.6 (5.9)	<0.001	23.8 (7.8)	34.4 (6.7)	<0.001	*p* < 0.001	*p* = 0.97

Patient data were divided into four groups according to SVA and abdominal circumference. SVA: sagittal vertical axis, SS: SVA < 40 mm and abdominal circumference < 80 cm, LS: SVA < 40 mm and abdominal circumference ≥ 80 cm, SR: SVA ≥40 mm and abdominal circumference < 80 cm, LR: SVA ≥ 40 mm and abdominal circumference ≥80 cm, BMI: body mass index, SMI: skeletal muscle mass index, CCI: Charlson comorbidity index, CONUT: Controlling Nutritional Status, PT: pelvic tilt, SD: standard deviation.

**Table 3:** The results of age-adjusted logistic regression analysis.

Factor	Odds Ratio (95%CI) for High Fall Risk Score	*p*-Value
Sarcopenia	3.27 (1.47–7.29)	*p* < 0.01
SVA (mm)	1.01 (1.01–1.02)	*p* < 0.001
Abdominal circumference (cm)	1.04 (1.00–1.07)	*p* < 0.05

CI: confidence interval, SVA: sagittal vertical axis.

## Data Availability

The datasets used and analyzed in the current study are available from the corresponding author upon reasonable request.
